# Brain leukocyte infiltration initiated by peripheral inflammation or experimental autoimmune encephalomyelitis occurs through pathways connected to the CSF-filled compartments of the forebrain and midbrain

**DOI:** 10.1186/1742-2094-9-187

**Published:** 2012-08-07

**Authors:** Charlotte Schmitt, Nathalie Strazielle, Jean-François Ghersi-Egea

**Affiliations:** 1 Inserm U1028, CNRS NMR5292, Lyon Neuroscience Research Center, Neurooncology & Neuroinflammation Team, Lyon 1 University, Faculté de Médecine Laennec, 7 rue G. Paradin, Lyon, F-69008, France; 2 Brain-i, Lyon, France

**Keywords:** Brain cisterns, Choroid plexus, Macrophage, Multiple sclerosis, Neuroinflammation, Ventricle

## Abstract

**Background:**

Cerebrospinal fluid (CSF) has been considered as a preferential pathway of circulation for immune cells during neuroimmune surveillance. In order to evaluate the involvement of CSF-filled spaces in the pathogenesis of experimental autoimmune encephalomyelitis (EAE), a model of multiple sclerosis, we performed a time-course analysis of immune cell association with the CSF-containing ventricles, velae, and cisterns in two active models of this disease.

**Methods:**

Guinea-pig spinal cord homogenate-induced EAE in rat and myelin oligodendrocyte glycoprotein-induced EAE in mouse were used. Leukocyte distribution and phenotypes were investigated by immunohistochemistry in serial sections of brain areas of interest, as well as in CSF withdrawn from rat. Immune cells associated with the choroid plexuses were quantified.

**Results:**

Freund’s adjuvant-induced peripheral inflammation in the absence of brain antigen led to a subtle but definite increase in the number of myeloid cells in the extraventricular CSF spaces. In both rats and mice, EAE was characterized by a sustained and initial infiltration of lymphocytes and monocytes within forebrain/midbrain fluid-filled compartments such as the velum interpositum and ambient cisterns, and certain basal cisterns. Leukocytes further infiltrated periventricular and pericisternal parenchymal areas, along perivascular spaces or following a downward CSF-to-tissue gradient. Cells quantified in CSF sampled from rats included lymphocytes and neutrophils. The distinctive pattern of cell distribution suggests that both the choroid plexus and the vessels lying in the velae and cisterns are gates for early leukocyte entry in the central nervous system. B-cell infiltration observed in the mouse model was restricted to CSF-filled extraventricular compartments.

**Conclusion:**

These results identified distinctive velae and cisterns of the forebrain and midbrain as preferential sites of immune cell homing following peripheral and early central inflammation and point to a role of CSF in directing brain invasion by immune cells during EAE.

## Background

In multiple sclerosis (MS), circulating leukocytes cross the blood-brain and blood-cerebrospinal fluid barriers (BBB and BCSFB, respectively) to gain access to the CNS. The BBB localizes at the endothelium of brain microvessels and the BCSFB is formed by the epithelium of the choroid plexuses (CP). Experimental autoimmune encephalomyelitis (EAE) is commonly used as an experimental model of MS to study the pathophysiology of this disease or to evaluate the efficacy of new therapeutic strategies. EAE is characterized by intermittent periods of progressive weakness or paralysis, concomitant to episodes of leukocyte infiltration and demyelination in the CNS [1,2]. In the early stages of EAE, the primary events triggering the migration of immune cells across non-inflamed CNS barriers, which are the actual sites of immune cell entry into the brain, and the pathways of cell trafficking within the brain, are not fully elucidated.

Cerebrospinal fluid (CSF) has been proposed to constitute a predominant route of T-cell trafficking to and from the CNS during physiological neuroimmune surveillance in humans [3,4]. In MS, the involvement of CSF in immune cell infiltration is suggested by the predominant localization of focal demyelinated plaques in the periventricular tissue and diffuse white matter injury in subpial areas [5,6]. Similarly, a spatiotemporal analysis of the pathogenesis of murine EAE indicated that periventricular and superficial white matter structures are primary targets of early T-cell infiltration [7]. The constitutive expression of P-selectin and the chemokine CCL20 in CP have pointed out the BCSFB as a direct route of lymphocyte migration from the blood into the brain [8-10]. This route accounts for the lack of permissivity of the resting brain endothelium to immune cell migration during neuroinflammation initiation. Other studies rather proposed that the vessels running in subarachnoid spaces of the lower part of the spinal cord were the initial site of T-cell entry into the CSF during EAE in rat, implying that EAE is primarily a spinal cord disease [11].

The complexity of CSF circulation needs to be taken into account to fully understand immune cell trafficking into and within the brain. This fluid is produced by the CPs in both lateral, third, and fourth ventricles. It flows briskly through the ventricular system and more slowly through a network of velae as well as internal and ventral cisterns present in both rodent and human, before reaching the external subarachnoid spaces surrounding the brain. There, it partially mixes with the CSF filling the meningeal spaces of the spinal cord. A large part of CSF is contained in the cisterns of the forebrain and midbrain, which connect to the third and mesencephalic ventricles via the different velae [12-14].

In this paper we seek to evaluate the role of this CSF circulatory network in the intracerebral dissemination of different types of immune cells during the early phases of EAE. We established the spatiotemporal cerebral distribution of T-, B-lymphocytes, monocytes, and neutrophils. Two different models of EAE were analyzed to account for potential model-to-model variation in immune cell trafficking. We show in both models the systematic implication of CSF-filled spaces of the forebrain and midbrain in the initial distribution of immune cells in the CNS.

## Material and methods

### Animals

Ninety-three Dark Agouti (DA) female rats (130 to 140 g) were purchased from Janvier (Saint Genest sur Isle, France) and 30 C57BL/6 J female mice (8-week-old) from Charles River (Saint Germain sur l’Arbresle, France). They were maintained on a 12:12-h light/dark cycle. All animal research was conducted according to the French Ethical Committee guidelines (decree 87 to 848) and the European Community directive 86 to 609-EEC.

### Induction of experimental autoimmune encephalomyelitis or peripheral inflammation

EAE was induced in isoflurane-anesthetized DA rats by intraplantar injection of guinea-pig spinal cord homogenate (830 μg/paw), emulsified at 4°C in 100 μL of complete Freund’s adjuvant (CFA; Difco, Detroit, MI, USA) supplemented with 250 μg *Mycobacterium tuberculosis* (Difco). EAE was induced in isoflurane-anesthetized C57BL/6 J mice by injecting each flank subcutaneously with 50 μg of MOG_35-55_ (MEVGWYRSPFSRVVHLYRNGK; GeneCust, Luxembourg) in 100 μL CFA. *Bordetella pertussis* toxin (200 ng in 100 μL; Sigma, St Louis, MO, USA) was injected intravenously in mice, on the day of initial vaccination and 2 days later. Other animals were injected following the same protocol as for EAE-diseased animals but the brain antigen was omitted. They were considered as animals suffering from a peripheral inflammation. Animals were monitored daily, weighed, and the clinical score (CS) was determined as follows: CS1, tail weakness; CS2, tail paralysis; CS3, hindlimb weakness; CS4, hindlimb paralysis. When clinical signs were graded as intermediate between two scores, 0.5 was added to the lower value. On post-vaccination days 2, 4, 6, 9 (onset of the disease), and 11 (peak of the disease), rats were either sacrificed by decapitation following light anesthesia or perfused with 10 mL of 0.9% NaCl under i.p. pentobarbital anesthesia. All mice were anesthetized with an i.p. injection of pentobarbital on days 1, 8, 11 (onset of the disease), and 13 (peak of the disease), and were perfused with 5 mL of 0.9% NaCl prior brain sampling. Following cranial bone parting, brains were immediately removed, frozen in -45°C isopentane and stored at -80°C.

### CSF and blood sampling in rats

CSF was sampled from additional control, peripherally inflamed (PI), and EAE-diseased rats under isoflurane anesthesia as follows: the head positioned in a stereotaxic frame was tilted downward to expose the neck. Following skin incision, muscles were gently removed to uncover the cisterna magna. A dental needle (30 G) fixed to a holder and connected to a tubing was approached parallel to the bregma/lambda axis to collect the CSF through the cisterna magna. A minimum of 50 μL of CSF was sampled using a collection tubing precoated with bovine serum albumin (BSA). The collection tubing contained 5 μL of a phosphate buffer saline (PBS) solution containing 0.1% BSA that was flushed after sampling to ensure full recovery of the collected CSF. CSF was then centrifuged for 10 min at 800 *g* at 4°C. Most of the CSF was then removed and the remaining 10 μL of CSF containing the cells was spread on a slide and left to dry at 37°C, prior to acetone/methanol (1/1) fixation for 2 min at room temperature. Slides were stored at -20°C until immunocytochemical analysis. Samples containing red blood cells were discarded from the analysis.

Following CSF sampling, animals were sacrificed and blood was collected in heparinized tubes. A total of 200 μL of blood underwent red blood cell lysis in an ammonium chloride potassium buffer (150 mM NH_4_Cl, 10 mM KHCO_3_, 0.1 mM Na_2_EDTA, pH 7.4) for 5 min. Samples were diluted with PBS and centrifuged at 400 *g* for 5 min. Drops of resuspended leukocytes were left to dry on slides prior to fixation in acetone/methanol for 2 min and stored at -20°C for immunocytochemistry.

### Immunohistochemistry and immunocytochemistry

Seven μm-thick sections of rat and mouse frozen brain were cut from areas selected to conduct an extensive analysis of immune cells in the ventricular and extraventricular CSF-containing compartments and in the adjacent neural structures. The respective location of these different spaces is illustrated in Additional file 1. The ventricular compartments included, in a rostro-caudal order and following the flow of CSF, the anterior and posterior horns of the lateral ventricle, the dorsal and ventral part of the third ventricle, the aqueduct and mesencephalic ventricle, and the central part and lateral recesses of the fourth ventricle. The internal extraventricular compartments included the velum interpositum pouches abutting the dorsal part of the third ventricle and pineal recess, the quadrigeminal and ambient cisterns separating the midbrain from the cortices, the superior medullary velum abutting the mesencephalic ventricle, and the ventrolateral cerebellar meninges. The more external extraventricular compartments included, at the base of the brain and in a caudo-rostral order, the interpeduncular fossa, the cistern of the optic tract, and the cistern of the laminae terminalis, as well as the rhinal fissure at the surface of the cortex. The leptomeninges at the base of the brain and at the surface of the cortex and cerebellum were also examined when they remained adherent to the extracted brains. In each selected area, two sets of sections separated by 100 μm were analyzed to replicate the observations. Frozen brain sections sampled on Superfrost Plus microscope slides (Menzel, Braunschweig, Germany) were dried at room temperature. Sections were fixed without rehydration. Fixation was performed in acetone/methanol for 5 min or in ethanol for 10 min for rat or mouse tissue, respectively. Both the use of Superfrost Plus slides and the fixation protocol were chosen to minimize the loss of free floating cells. The fixation procedures were also found optimal for the antibodies used in each species.

Fixed brain sections or cells isolated from blood or CSF were blocked for 1 h at room temperature in PBS supplemented with 0.2% BSA, 0.8% normal goat serum, and for CD45 antibody with 0.2% Triton X-100. Immune cells were detected and characterized in rat using mouse monoclonal antibodies against CD45 (panleukocyte, cat. 550556 - 0.625 μg/mL), CD45RA (B lymphocytes, cat. 554882 - 2.5 μg/mL), both from BD Pharmingen (Le Pont de Claix, France), CD68 (monocyte/macrophages, cat. MCA341R - 1.25 μg/mL) from AbD Serotec (Colmar, France) and rabbit anti-CD3 (T lymphocytes, cat. ab5690 - 1 μg/mL) from Abcam (Cambridge, UK). Rat monoclonal antibodies against CD3 (T cells, cat. 555273 - 1.6 μg/mL), CD45R/B220 (B cells, cat. 01121D - 1.6 μg/mL) from BD Pharmingen and CD68 (monocyte/macrophage, cat. MCA1957 - 1.25 μg/mL) from AbD Serotec were used on mouse tissue sections. A rabbit polyclonal antibody against myeloperoxidase (MPO) (cat. A0398 - 1 μg/mL, Dako, Glostrup, Germany) was used for double staining with CD68 in both species. It strongly labels neutrophils, and to a lower extent some macrophages. Sections or cells were incubated with the primary antibodies overnight at 4°C in blocking buffer, and then rinsed four times in PBS. Sections or cells were incubated with secondary Alexa Fluor 488 or 555-conjugated goat anti-mouse, anti-rat, or anti-rabbit antibodies (Invitrogen, Cergy Pontoise, France) for 1 h at room temperature. In some experiments, laminin staining (cat. 24851 - 12.5 μg/mL, (Novotec, St Martin La Garenne, France) was performed in double-labeling to visualize the vessels. Nuclei were stained with 0.1 μg/mL DAPI (Roche Diagnostics, Mannheim, Germany) for 5 min. Images were acquired under epifluorescence illumination (at 488 and 555 nm) with an AxioVision microscope (Zeiss, Jena, Germany). The number of immune cells in blood and CSF was expressed as a percentage of immunolabeled cells relative to the total number of cells assessed by DAPI.

TUNEL staining was realized on brain sections using the DeadEnd^TM^ Fluorimetric TUNEL System (Promega, Madison, WI, USA). Positive staining was obtained by pretreating sections with DNAse I, according to the manufacturer’s protocol.

### Immune cell quantification in choroid plexuses

Rat CD45^+^ cells and mouse CD3^+^ and CD68^+^ cells were quantified in both the lateral and fourth ventricle CP as a percentage of immune cells relative to the total number of cells in the choroidal tissue as follows. Four brain sections were analyzed for each CP. Two sections were selected 50 μm apart around bregma -3.3 mm and two others around bregma -3.8 mm at the level of the lateral ventricle. Sections were chosen at bregma -11.5 mm and bregma -12 mm at the level of the fourth ventricle. CPs were manually delimited on the micrographs and the total number of cells (based on DAPI staining) and that of immune cells were quantified using the software ImageJ.

## Results

### Clinical and biological courses of control, PI, and EAE-diseased animals

Peripheral inflammation and EAE were induced in four independent batches of rats and in two independent batches of mice. In rat, both PI and EAE-diseased animals displayed a sustained paw inflammation throughout the experimental period. PI and EAE rats and mice gained less weight than control animals from D1 post-injection and throughout the experimental period. Concomitantly to the appearance of clinical signs, that is, at P9 in rat and P8 in mice, EAE-diseased animals started to lose weight (see Additional file 2 for more details).

**Figure 1:**
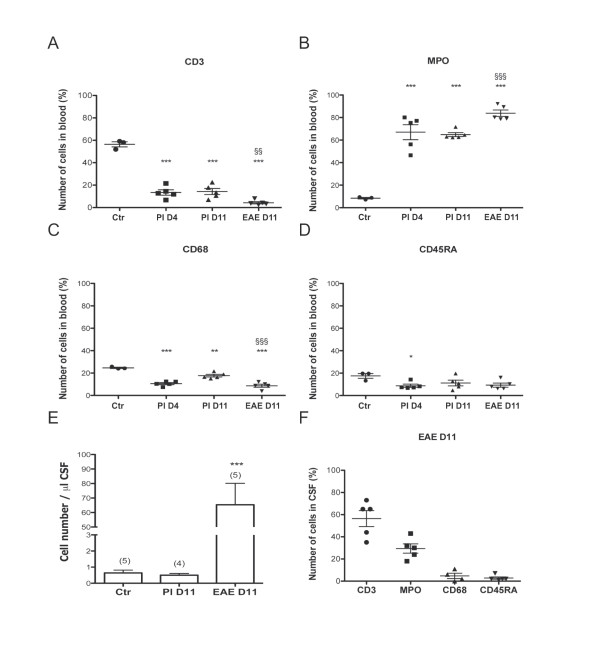
**Phenotypic analysis of leukocytes in blood and CSF from control, PI, and EAE-diseased rats. (A, B, C, D)** Immunocytochemical identification of blood leukocytes. The percentages of CD3^+^ T cells, CD68^+^ monocytes, and to some extent CD45RA^+^ B cells decrease, while the percentage of MPO^+^ neutrophils increases strongly in both PI and EAE animals in comparison to the control group (Ctr), (Dunnett’s test, * *P* < 0.05, ** *P* < 0.01, *** *P* < 0.001). The percentages of MPO^+^, CD3^+^, and CD68^+^ cells in the blood are also statistically different between PI animals and animals with EAE at day 11. One-tailed *t*-test for unequal variance, §§ *P* < 0.01, §§§ *P* < 0.001. **(E)** Few cells are present in the CSF of control and PI rats. The number of cells increases significantly in the CSF of EAE-diseased animals. Dunnett’s test, *P* < 0.001 (***). **(F)** The main population of cells in the CSF of EAE-diseased rats is CD3 positive and a significant proportion of MPO^+^ cells is also present. In A-D and F, the percentages of leukocytes that were CD3^+^, MPO^+^, CD68^+^, CD45RA^+^ cells, are given for individual animals, with the mean ± SEM. In E, data are expressed as mean ± SEM, (*n*).

T cells identified as CD3^+^ cells amounted to 60% of total leukocytes in the blood of control rats (Figure 1A), while MPO^+^/CD68^-^ neutrophils represented less than 10% of leukocytes (Figure 1B). In the blood of PI and EAE-diseased animals, the ratio of CD3^+^ cells over MPO^+^ cells was reversed with neutrophils amounting to 60% of total leukocytes, both early after induction and at the peak of EAE (Figure 1A and B). CD68^+^ cell counts revealed a significant decrease in the proportion of monocytes in the blood of PI and EAE-diseased animals, compared with control animals (Figure 1C). Similarly, the number of CD45RA^+^ B cells decreased in the blood of PI animals sacrificed on D4 (Figure 1D).

Cell counts in CSF were very low in both control and PI animals (less than 1 cell/μL CSF), and strongly increased in EAE-diseased animals during the clinical phase of the disease (Figure 1E). In control and PI animals CSF leukocytes were almost exclusively CD45^+^, CD3^+^ cells (data not shown). In EAE-diseased animals, CSF-borne cells comprised 30% MPO^+^ neutrophils, and a few percents of CD45RA^+^ B cells and CD68^+^ monocytes in addition to the main CD3^+^ cell population (Figure 1F).

### Distribution and phenotypic analysis of immune cells in the brain of control and PI rats

**Figure 2:**
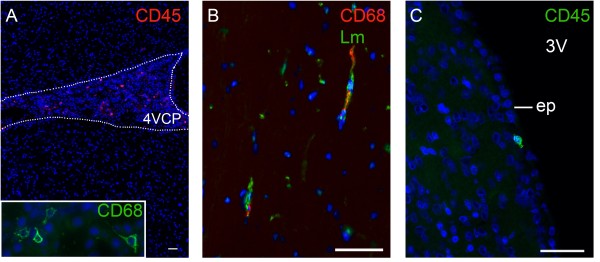
**Immune cell localization in parenchymal and ventricular compartments in the brain of control rat. (A)** Leukocytes are preferentially associated with the CPs (illustrated here in the fourth ventricle). A high magnification is shown in the insert. **(B)** Leukocytes in brain parenchyma are CD68^+^ cells associated with perivascular spaces. Blood vessels are visualized by laminin. **(C)** Occasional cells not associated with blood vessels are seen in a subependymal position. Scale bar, 50 μm. 3 V, third ventricle; ep, ependyma; Lm, laminin.

**Figure 3:**
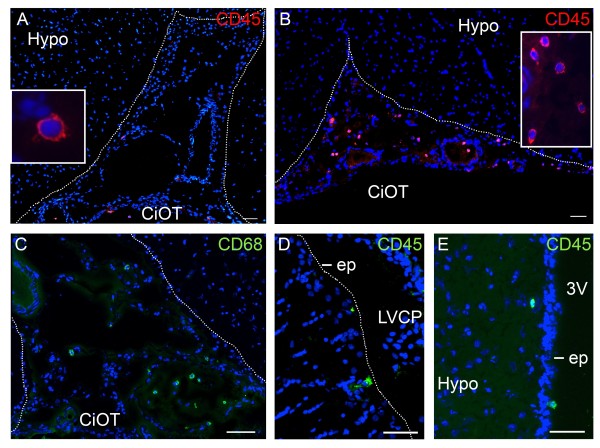
**Cisternal and subependymal localization of immune cells in the brain of PI rats. (A, B, C)** The number of cisternal immune cells increases in PI rats (B, illustrated by the optic tract cistern at D11) in comparison to control (A) animals. Most of these cells are CD68^+^ monocytic cells (C). **(D, E)** In PI rats, leukocytes are also observed in a subependymal position. CD45^+^ immune cells at the level of the lateral (D) and third (E) ventricles are shown. Inserts in panel A and B show higher magnifications of immune cells. Scale bar, 50 μm. Dashed lines delimit cisterns (A, B, C) or ventricles (D, E). 3 V, third ventricle; CiOT, cistern of the optic tract; ep, ependyma; Hypo, hypothalamus; LVCP, lateral ventricle choroid plexus.

Only a limited number of CD45^+^ cells were detected in the brain of control rats. The highest density of these cells was found in the CPs (Figure 2A), and the majority of them were CD68 positive. Immune cells, mainly CD68^+^ cells, were also preferentially located in the velum interpositum and ambient cisterns, as well as externally in the basal cisterns, basal meninges, and in the largest cerebellar meningeal infoldings (for example, Figure 3A for the cistern of the optic tract). All anatomical locations are shown in Additional file 1). Occasional CD68^+^ cells were observed in certain circumventricular organs, that is the subfornical organ, the median eminence, and the area postrema. Very few CD68^+^ cells were present in the brain parenchyma. They were associated with microvessels (Figure 2B), visualized with an anti-laminin antibody that labels the basal membrane of the microvasculature [15]. Those cells were still found in animals perfused with physiological saline prior to brain sampling, and were thus identified as resident perivascular cells. Perfusion did not change the localization pattern of immune cells in CPs or cisternal spaces, with the exception that it cleared them from the lumen of large vessels, confirming that immune cells associated with these spaces are resident cells. Finally, a limited number of cells (two to three per section) that did not associate with laminin, were found in the neural tissue in close proximity to the cisterns of the velum interpositum, to the ependyma that borders the lateral and third ventricles (Figure 2C), or to the margin of the lateral recesses of the fourth ventricle.

In PI animals, the number of cells present in the brain remained low, but subtle changes could be observed in several CSF-containing spaces compared with control animals. While the number of CD45^+^ cells remained steady in the CPs (see Additional file 3), it progressively increased from D2 up to D11 in the cistern of the velum interpositum, in quadrigeminal and ambient cisterns, and in cisterns of the optic tracts, in 60% to 70% of PI animals (Figure 3A and B, Additional file 1). The phenotypic analysis of the cells present in the velum interpositum and ambient cisterns of perfused PI animals at D6 and D11 revealed a majority of CD68^+^ cells (Figure 3C), and a small proportion of MPO^+^ and CD3^+^ cells (data not shown). As in control rats, a few immune cells were observed in an ependymal/subependymal position at the level of the lateral, third and fourth ventricle (Figure 3D and E). Brain parenchymal regions, including the cortex, hippocampus, hypothalamus, and areas more prone to leukocyte infiltration during EAE (see below), seldom contained immune cells and were not different from those observed in control animals.

### Distribution of immune cells in the brain of EAE-diseased rats

**Figure 4:**
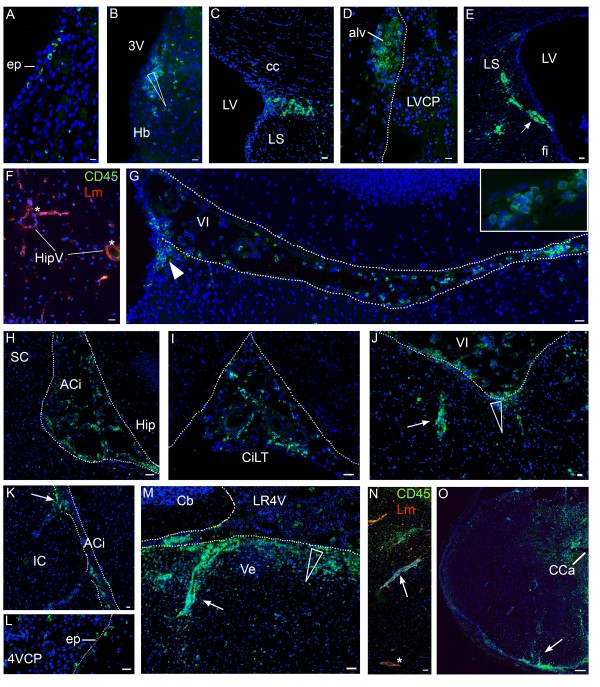
**Distinctive localization of immune cells in the CNS of EAE-diseased rats. (A to K)** CD45^+^ immune cells in the forebrain. **(L to O)** CD45^+^ cells in the hindbrain. **(A)** CD45^+^ cells are visualized in a subependymal location at D9. **(B)** (D9), **(C to F)** and **(L to N)** (D11). CD45^+^ cells are observed in certain periventricular areas. Cells often infiltrate the tissue as clusters in the habenula (B), ventrally to the corpus callosum **(C)**, in the hippocampal alveus close to the lateral ventricle **(D)**, in the pons along the fourth ventricle **(L)** and its recesses **(M)**. Cells infiltrate the tissue in a disseminated manner following a downward density gradient from the CSF to the parenchyma (B,M, arrowheads) or along perivascular spaces close to the ventricle, for example in the fimbriae **(E)**, and in the fourth ventricle (M,**N**, arrows). There are no cells around vessels in deeper structures such as the hippocampal fissure (star in **(F)**) and the pons (star in (N)). Laminin (lm) stains the basal membrane of the vessels. In (N) the upper side of the image is facing CSF; the lower side is deep pons tissue. **(G to K)** By D11, the number of leukocytes has increased in extraventricular CSF-containing internal compartments such as the velum interpositum (G, **J**) and ambient cisterns **(H, K)** and also in more peripheral cisterns such as the cistern of the laminae terminalis **(I)**. The insert in (G) shows a high magnification of CD45^+^ cells in the velum interpositum space. Some infiltrates are localized between the third ventricle and the cistern of the velum interpositum (G, white arrowhead). Cell infiltrates displaying a downward CSF to tissue density gradient are found ventrally to the velum interpositum (arrowhead in (J)) or along perivascular spaces adjacent to extraventricular CSF-filled compartments (J, K, arrows). (O) In spinal cord leukocytes massively infiltrate the meninges and adajcent perivascular spaces (arrow). They also disseminate in the parenchyma, dorsally to the central canal. Scale bar, 20 μm (A to L), 50 μm (M to O). Dashed lines delimit ventricles (C, M) or cisterns (G to K). 4VCP, fourth ventricle choroid plexus; ACi, ambient cistern; Cb, cerebellum; cc, corpus callosum; CCa, central canal; CiLT, cistern of the laminae terminalis; ep, ependyma; fi, fimbria; Hb, habenula; Hip, hippocampus; HipV, hippocampal vessels; IC, inferior colliculus; LS, lateral septum; LV, lateral ventricle; LVCP, lateral ventricle choroid plexus; SC, superior colliculus; Ve, vestibular nucleus (pons); VI, velum interpositum cistern.

**Figure 5:**
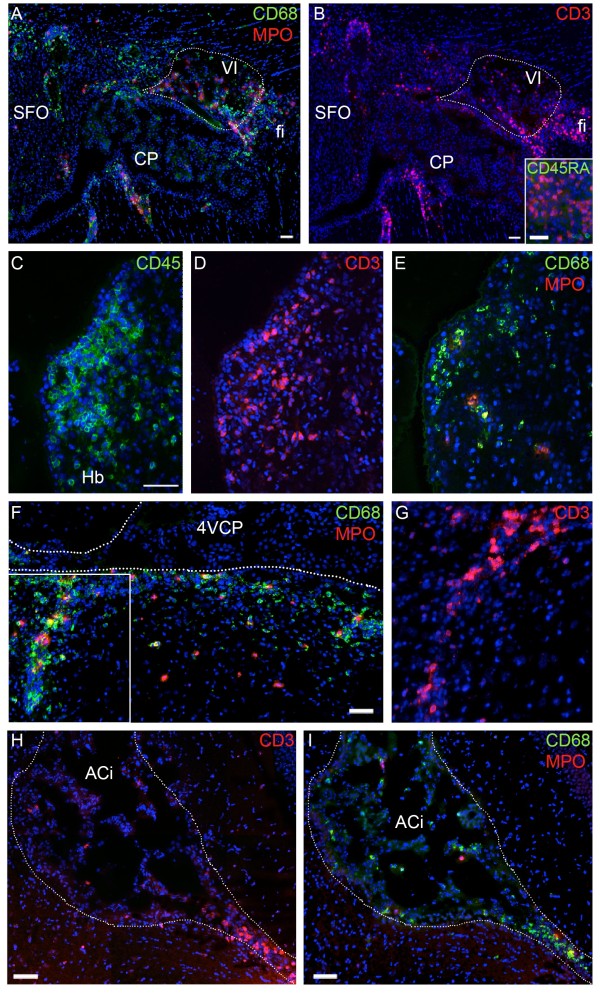
**Phenotypic analysis of immune cells infiltrating the CSF spaces and the brain parenchyma of rats during EAE. (A, B)** CD68^+^, MPO^+^ (A), CD3^+^ (B) and CD45RA^+^ (B insert, green) cells are shown at D9 in the area of the subfornical organ. CD68^+^ monocytic cells and CD3^+^ T cells form the majority of cells in the infiltrates. A significant proportion of MPO^+^ neutrophils and very few CD45RA^+^ B cells are also observed. Note the limited number of immune cells associated with the subfornical organ. **(C to I)** Identification of the cell types infiltrating the brain at D11. The two main populations of immune cells remain CD3^+^ and CD68^+^ cells but the proportion of these two cell types vary from cluster to cluster. (C) shows a periventricular parenchymal cell cluster in the forebrain, which is mostly formed by CD3^+^ cells **(D,E)**. Infiltrating immune cells in the periventricular medulla close to the CP of the fourth ventricle spread along a vessel **(F)**. These cells are monocytes/macrophages (F), as well as CD3^+^ cells (**(G)**, corresponding to the rectangle in (F)). A large number of CD3^+^ and CD68^+^ cells infiltrate the internal CSF cisterns as shown at the level of the ambient cistern **(H,I)**. The number of neutrophils remains very limited in both parenchymal and CSF spaces (E, F, I). Scale bar, 50 μm. Dashed lines delimit ventricles (F) or cisterns (H,I). 4VCP, fourth ventricle choroid plexus; ACi, ambient cistern, fi, fimbria; Hb, habenula; SFO, subfornical organ; VI, velum interpositum cistern.

During EAE, from D2 to D6 post-induction, immune cell distribution in the velae and cisterns of the brain was similar to that observed in the brain of PI rats (data not shown). When clinical neurological signs became apparent, various drastic changes in cell distribution were observed, with the notable exception of the CPs in which the number of associated leukocytes did not vary throughout the post-induction period (see Additional file 3). At D9 a few leukocytes were observed in the ventricular system of the forebrain and midbrain (not shown). Individual cells or small clusters of cells were observed more frequently in a subependymal position than at earlier times. This localization suggests that these leukocytes originate from the CSF (Figure 4A). Immune cells infiltrated the periventricular habenula (Figure 4B) and the fimbria in several animals. At D11 clusters of immune cells were observed in two-thirds of the animals in the tissue adjacent to the upper corner of the lateral ventricle anterior horn and ventral to the corpus callosum (Figure 4C). Leukocyte infiltrates were also observed in periventricular structures such as the hippocampal alveus bordering the lateral ventricle (Figure 4D), the fimbria/stria terminalis area, the thalamus ventral to the anterior horn of the lateral ventricle, the region between the dorsal and ventral part of the third ventricle, and most typically the habenula (for example, Figure 5C). Cells either formed penetrating clusters adjacent to perivascular spaces (Figure 4E, arrow), or disseminated into the tissue following a downward density gradient from the ventricular compartment (Figure 4B, arrowhead). No cell was observed in deeper brain structures, even when obvious large perivascular spaces were present, such as along the hippocampal fissure (Figure 4F). Cell clusters were often present at the interface between the dorsal part of the third ventricle and the velum interpositum pouches (Figure 4G, arrowhead).

Prominent infiltrates were also observed in extraventricular CSF-filled pockets and adjacent structures (Additional file 1) of EAE-diseased animals. From D9, CD45^+^ cells in the velum interpositum, quadrigeminal, and ambient cisterns, and in the cisterns of the optic tract were in larger numbers than in PI animals, sometimes distributing as small clusters, but never forming clear perivascular cuffs around cisternal vessels. At D9, the number of cells in these spaces was not dependent on the clinical score of the animals. At the peak of the disease (D11, Figure 4G, H), immune cell invasion in these extraventricular CSF spaces increased even further, and extended both rostrally to the cistern of the laminae terminalis (Figure 4I) and adjacent meninges, and caudally to the interpeduncular fossa and surrounding ventral meninges. It did not, however, extend up to the rhinal fissure, the cortical meninges, or the superior medullary velum. Cells often accumulated along the glia limitans, a thick layer that borders the midbrain structures facing the quadrigeminal and ambient cisterns [12]. From these latter CSF-filled compartments, they hardly infiltrated the tissue (left of dashed lines Figure 4H,K) unless they followed specific large periventricular spaces around penetrating vessels (arrow in Figure 4K). TUNEL staining indicated that the immune cells present in these extraventricular CSF spaces were not apoptotic (Additional file 4). Parenchymal infiltrates were observed in other distinctive regions in close vicinity of extraventricular CSF spaces. These areas included the white matter forming the ventral hippocampal commissure and fimbria adjacent to the pouches of velum interpositum (Figure 5A,B), as well as the brain structures ventral to the velum interpositum including the habenula. This structure was infiltrated in all animals at the peak of the disease (Figure 4J). In the basal midbrain, immune cells were seen either along penetrating vessels dorsal to the cisterns of the optic tract, but not in the optic tract itself, or infiltrating the tissue on each side of the optic chiasma and median eminence.

As for the hindbrain a few immune cells were present in CSF within the fourth ventricle throughout the post-induction period, and their number increased at D11. At that time, occasional cells were seen in an immediate subependymal location in the central part of the fourth ventricle (Figure 4L) or associated with the glia limitans bordering the inferior cerebellar peduncles along the lateral recesses. At D11 numerous cells were also observed deeper in the periventricular tissue at the level of both the central and lateral part of the fourth ventricle and its recesses (Additional file 1). They infiltrated following a downward CSF-to-tissue density gradient or formed perivascular cuffs around blood vessels in the vicinity of the ventricular CSF (Figure 4M). Cell infiltrates around perivascular spaces were also seen in the vicinity of basal meninges at D11 (not shown). Perivascular spaces of deep vessels in the pons (Figure 4N), medulla oblongata, and cerebellum were spared, and infiltration of the basolateral cerebellar meninges was not observed or limited. In general the cerebellum did not display any inflammatory involvement, with only occasional increased numbers of immune cells in the large sulci of this structure. Immune cells were numerous in the meninges surrounding the spinal cord, and displayed a spreading pattern following a downward gradient from the surface to the central core of the cord, either as scattered parenchymal cells or along radially oriented perivascular spaces. The region dorsal to the central canal was prominently invaded (Figure 4O).

### Phenotypic analysis of immune cells in the brain of EAE rats

The phenotype of immune cells invading the brain during EAE was investigated in more details. All monocytes/macrophages, T cells, B cells, and neutrophils were found in cisternal compartments and in tissue infiltrates, albeit in different proportions. At the onset of neurological signs, the majority of leukocytes were CD68^+^ or CD3^+^ cells (Figure 5A, B). A significant number of MPO^+^ cells was already present (Figure 5A). Very few cells were CD45RA^+^ positive (Figure 5B, insert). By D11, cells that were present in periventricular clusters (Figure 5C to G) and in cisterns (Figure 5H, I) were mostly CD3^+^ or CD68^+^ cells, the proportion of these two cell types varying among clusters. Both cell types were found to disseminate from the periventricular clusters into the tissue or along perivascular spaces (Figure 5D, F, G).

Finally, despite the large infiltrates observed in periventricular areas in the vicinity of some circumventricular organs during EAE (Figure 5A, B), the number of immune cells within these organs was only marginally increased in comparison with PI rats. Cells were never present in the subcommissural organ of any animal.

### Distribution and phenotypic analysis of immune cells in the brain of control, PI, and EAE-diseased mice

**Figure 6:**
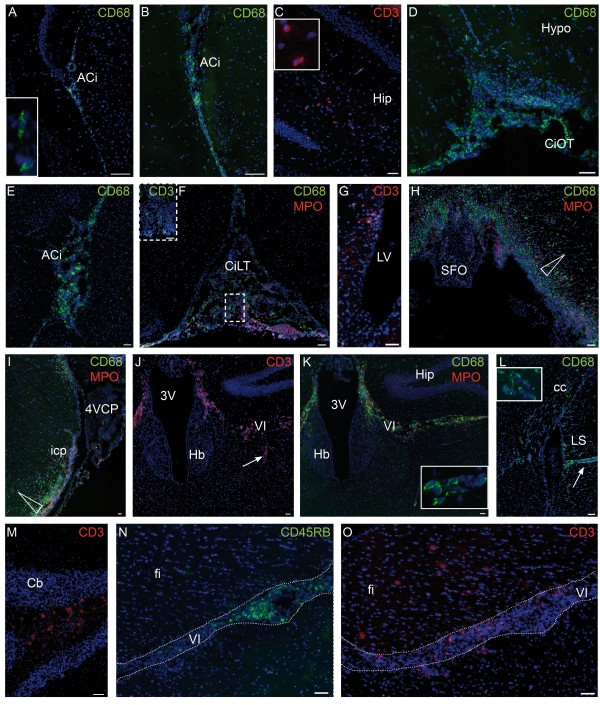
**Distinctive localization of immune cells in the CNS of EAE-diseased mice. (A)** Localization of leukocytes in the brain of control mice. Leukocytes are mainly CD68^+^ monocytic cells and preferentially localize in basal and internal cisterns of the brain such as the ambient cistern. **(B,C)** Increase in leukocyte number in the brain of PI animals. At P10-14, the number of CD68^+^ cells slightly increases in these CSF compartments (B), and some leukocytes, including CD3^+^ T cells infiltrate the hippocampus (C). **(D to O)** Immune cell infiltration in the brain of EAE-diseased animals. **(D, F, H, L)** are from animals with a clinical score of 1, and **(E, G, I to K, M to O)** are from animals with a clinical score of 4. Similar observations were made for both scores. The number of CD68^+^ cells increases drastically in the extraventricular CSF compartments such as the cisterns of the optic tract (D), the interpeduncular and ambient cisterns (E), and the velum interpositum space (K). Leukocytes in extraventricular spaces spread rostrally to the cistern of the laminae terminalis (F). CD3^+^ T cells and MPO^+^ neutrophils are also present in these spaces (F, dashed insert in F, **J**, K). Immune cells infiltrate the parenchyma in subependymal locations (G), and in forebrain and midbrain structures in close proximity to the CSF (H, J, K, **L**). On the caudal side, infiltrates reach the structures that border the lateral recesses of the fourth ventricle such as the inferior cerebellar peduncles (**I**). Tissue infiltration occurs following a downward CSF-to-tissue gradient (arrowhead in **H** and I) or along perivascular spaces (arrow in J and L). Tissue infiltrates are composed mainly of CD3^+^ (J) and CD68^+^ cells (H, I, K). To a lesser extent MPO^+^ cells are also present (H, I, K). The mouse EAE model is characterized by the additional infiltration of CD68^+^ and CD3^+^ cells in the white matter of the corpus callosum (L) and of the cerebellum **(M)**. B cells are found in CSF-filled cisterns **(N)** in the same proportion as T cells, but do not invade the parenchyma as do CD3^+^ cells (O). In (A, C, K, L) inserts show high magnifications of immune cells in the areas of interest. Scale bar, 50 μm (A-H, J-O), 100 μm (I). 3 V, third ventricle; 4VCP, fourth ventricle choroid plexus; ACi, ambient cistern; Cb, cerebellum; cc, corpus callosum; CiLT, cistern of the laminae terminalis; CiOT, cistern of the optic tract; fi, fimbria; Hb, habenula; Hip, hippocampus; Hypo, hypothalamus; icp, inferior cerebellar peduncle; LS, lateral septum;LV, lateral ventricle; SFO, subfornical organ; VI, velum interpositum cistern.

Brain sections from control mice were subjected to a thorough search of immune cells using antibodies raised against CD3 and CD68. T cells identified as CD3^+^ cells were scarce and did not display any preferential localization in the parenchymal, cisternal, or meningeal spaces. CD68^+^ monocytic cells were more abundant than CD3^+^ cells. As seen in rat, they were found preferentially in the cisterns of the velum interpositum, the cisterns of the optic tract, and the ambient cisterns (Figure 6A), as well as in all CPs (shown in Additional file 3). In brain parenchyma the CD68 antibody also labeled microglial cells, recognizable by their specific morphology (not shown). Based on that discriminating criterion, non-microglial CD68^+^ cells were rare in the neuropil, with no difference between the rostral and caudal parts of the brain.

In PI mice, no difference in the number and repartition of immune cells was observed at D1 in comparison with control animals. In one out of three mice sacrificed at D8 and in all mice sacrificed at D11 to D13 an increased number of CD68^+^ cells and some CD3^+^ cells were observed in the cisterns of the velum interpositum, the cisterns of the optic tract and the ambient cisterns in the forebrain (Figure 6B). At the latest time, this observation extended to the meninges at the base of the brain and the cistern of the laminae terminalis (not shown). This increase in cell number was more prominent in the rostral than in the caudal part of the brain. Half of these PI mice also had CD68^+^ cells, and to a lesser extent MPO^+^ neutrophils and CD3^+^ cells infiltrating the hippocampus without forming cell clusters (Figure 6C). No change in the morphology or staining pattern of microglia was seen in PI animals, and B cells were absent from their brain. In both control and PI animals, occasional CD68^+^ immune cells were observed in a subependymal localization (not shown).

The quantification of immune cells associated with the CP stroma of all control, PI, and EAE-diseased mice revealed no difference in CD68^+^ cell numbers between groups, and only a modest increase of CD3^+^ cells in PI and EAE-diseased animals compared with controls (see Additional file 3). By contrast, all mice subjected to EAE presented on D8, prior to neurological signs, an increased number of both CD68^+^ and CD3^+^ cells in several CSF-filled spaces by comparison to PI animals. These spaces included the velum interpositum pouches, the cisterns of the optic tract, the interpeduncular and ambient cisterns, and the meninges at the base of the brain. At the onset of the neurological disease, cell numbers increased even further in the CSF-containing cisterns of the forebrain and midbrain (Figure 6D, E, J). MPO^+^ neutrophils were also present (Figure 6K). Infiltrates extended up to the more rostral cistern of the laminae terminalis (Figure 6F). Immune cells were also observed in the ventricular CSF and in subependymal locations (Figure 6G). Scattered CD68^+^ and CD3^+^ cells, and in a lower proportion MPO^+^ cells, disseminated in the hippocampal commissure and fimbria close to the rostral pocket of the velum interpositum in the vicinity of the subfornical organ (Figure 6H). They also invaded as clusters, other distinctive areas adjacent to CSF-filled compartments such as the thalamic areas bordering the third ventricle and the velum interpositum, the inferior cerebellar peduncles (Figure 6I), and the peripheral spinal cord. Immune cells disseminated in the tissue along a downward gradient from the CSF spaces to the deeper structures (Figure 6H, I arrowhead), as well as along perivascular spaces (Figure 6J, L, arrows). The size of cell infiltrates in CSF-filled compartments and the distribution of cell clusters in the brain parenchyma were similar in mice with clinical scores of 1 or 4. CD68 staining was more intense in microglial cells located in the vicinity of infiltrates, suggestive of an activated phenotype for these resident cells (not shown).

The murine EAE model differed from the rat one by the additional presence of infiltrates within discrete white matter structures such as the part of corpus callosum bordering the lateral ventricle (Figure 6L), the optic tract along the optic cistern, and the cerebellar tracts (Figure 6M). It also differed by the absence of infiltrates in the habenula, a peculiar observation that would require confirmation in a larger number of animals (Figure 6J, K). Another characteristic of the mouse EAE model was the presence of B cells in CSF-filled spaces in the same proportion as T cells. However, in contrast to T cells, B cells were confined to these spaces, and were absent from parenchymal clusters as observed at the level of the fimbria (compare Figure 6N with 6O), or more caudally in the cerebellar white matter.

Finally, immune cells, mostly CD68^+^ cells, could be only occasionally observed in the subfornical organ, median eminence, and area postrema in control and PI mice. Their number was marginally increased in animals suffering from EAE, except in the area postrema where the density of CD68^+^ cells was higher and few CD3^+^ cells were observed. This was in contrast with the prominent infiltrates observed in the vicinity of these circumventricular organs in EAE-diseased animals (Figure 6H).

## Discussion

The present data describe the involvement of distinctive CSF-filled compartments as sites of immune cell homing and circulation within the CNS, following peripheral inflammation, and during the course of EAE.

### Myeloid cell recruitment to the brain following peripheral inflammation

Clinical and biological courses indicate that both PI and EAE-diseased animals undergo a sustained peripheral inflammation, throughout the 12 days of the experiment. In both PI rats and mice, a modest but consistent increase in the number of immune cells, mainly monocytes/macrophages, is observed in the velum interpositum cisterns and other cisterns located in the forebrain and midbrain, by comparison to control animals. This recruitment takes several days to build up. It shows that a sustained peripheral insult, such as that initiated by CFA, impacts on the innate neuroimmune surveillance. It also indicates that immune cells other than the previously described memory T-cells patrolling the CSF [9], are involved in this surveillance. In line with this finding are the increased number of monocytes observed in periventricular areas of animals suffering from a sustained hepatic inflammation [16], as well as the recruitment of neutrophils into CSF-filled spaces in the brain of mice enduring a severe systemic lipopolysaccharide-induced inflammation [17].

### Velae and cisterns of the forebrain and midbrain as homing sites for immune cells in the early stages of EAE

In EAE-diseased animals, immune cells infiltrate the same specific velae and cisterns of the forebrain and midbrain as in PI animals, but their number largely increases in these locations. This is perceptible just before neurological signs appear in mouse, or at the first occurrence of these signs (score 1 to 2) in rat. At later stages, immune cells spread to the more rostral cistern of the laminae terminalis and to caudal cisterns, and are found in distinctive areas of the surrounding neuropil as well as in the tissue lining the lateral recesses of the fourth ventricle. These data show that although EAE is often considered a spinal cord disease [11,18], forebrain and midbrain structures are subjected early to leukocyte infiltration, a feature shared by MS. The data also highlight the previously ignored involvement of the velum interpositum and ambient cisterns, and other cisterns of the forebrain and midbrain in the cerebral distribution of immune cells during EAE (Additional file 5). Undergoing a lower flow rate than the ventricles [12,14], these internal CSF-filled pockets through which major vessels such as choroidal arteries and internal cerebral veins are running, appear to be preferential niches for monocyte and T-cell homing to the brain. This also applies to B cells in the murine EAE model.

The limited information available concerning the membrane organization of these spaces is restricted to human. The basal cisterns such as the interpeduncular fossa or the cisterns of the optic tract share some anatomical similarities with the conventional external subarachnoid spaces, in particular the existence of arachnoid membranes trabeculae. Yet, they display a more complex compartmentalization supported by the presence of several layers of the Liliequist’s membrane [19]. The more internal CSF-filled spaces include the velum interpositum pouches which are closely associated with the choroidal tissue, and the ambient cisterns which in rodents contain a large proportion of the intracranial fluid supply (Ghersi-Egea and Fenstermacher, unpublished data). These spaces resemble tangles of pia, pial-based tela choroida, arachnoid membranes, and other membrane-forming cells of undefined origin [20-22]. Whether specific adhesion molecules and inflammatory modulators expressed by this membranous network and by the glia limitans forming the cisternal ventrolateral borders [12] are responsible for immune cell residence within these spaces remains to be investigated. The secretion of specific chemokines into the CSF by pericisternal and periventricular neural cells, including the choroidal epithelium [10,23,24], may also provide the appropriate chemoattractant environment for homing of activated monocytes and specific T-cells subsets to CSF-filled internal compartments.

Early infiltrating immune cells may enter CSF directly from the vessels present in the cisternal spaces and pouches of the velum interpositum, as it was shown for post-activated migratory T cells in the meningeal vessels at the surface of the spinal cord [11]. Extravasation mechanisms may however differ between the two sites. In the brain cisterns and velae, such infiltration presumably occurs only after vascular activation by proinflammatory factors or cells already circulating in CSF. Indeed, T cells, even pre-activated *in vitro*, fail to migrate across resting cerebral pial vessels [25]. Alternatively, in PI animals and during the early stages of EAE, immune cells may enter the ventricular CSF via the CP forming the BCSFB. This involves extravasation into the choroidal stroma and subsequent migration across the choroidal epithelial barrier into the fluid. Arguments in favor of this pathway include the presence of immune cells in ependymal or subependymal position in all control, PI, and EAE-diseased animals, and in the latters, the presence of infiltrates in periventricular areas. This distribution suggests that early infiltrating cells transit through the ventricular CSF and designate the CP as a possible gate of entry into the CNS. This hypothesis is further supported by data showing T lymphocytes and myeloid cells in the choroidal stroma this work [9,26] and by the constitutive expression of the adhesion molecules P- and E-selectins in choroidal vessels but not parenchymal vessels in human and healthy C57BL/6 mouse. Blocking strategies targeting P-selectin or its ligand PSGL-1 reduce early leukocyte infiltration [9,27].

### Phenotypic multiplicity and compartmentalization of immune cells in the brain during the early stages of EAE

The concurrent infiltration of CD68^+^ cells and CD3^+^ cells in CSF compartments of the forebrain and midbrain, and in periventricular tissues during the early phase of EAE in both animal models indicates that besides T lymphocytes, myeloid cells are also important effectors for disease initiation. A study on DA rats suffering from EAE also showed that the first infiltrating immune cells detected in the spinal cord were macrophages [28]. These cells have a stimulating potential towards T-cell responses, and are a major source of inflammatory and potentially cytotoxic mediators [29,30]. Together with resident dendritic cells they could support initial antigen presentation to penetrating T cells within the brain itself [28,31]. Macrophages and dendritic cells are also found in the choroidal stroma [26,32,33] and this study. They are however likely to present immunogens that differ from CNS antigens borne by the cisternal and meningeal cells, since the choroidal stroma lies outside the central compartment. This may explain why immune cells do not expand in CP during EAE. The limited increase in the number of T cells that we observed in the CP of PI and EAE-diseased mice likely reflects an enhanced T-cell transit from blood to CSF rather than a specific tropism for the choroidal tissue.

A large increase in the proportion of neutrophils among circulating leukocytes is observed in the blood of both PI and EAE-diseased animals. Neutrophils infiltrate the brain of EAE animals only, and can be found already in the early phase of the disease. In rat, most of them are found circulating into the CSF where they may be necessary for the effector phase of EAE pathogenesis [34]. This is in contrast to MS, in which neutrophils are seldom detected in CSF [35]. Finally, B cells, found especially in the mouse model are restricted to the CSF-filled cisterns, resembling the B-cell follicles detected in meninges of patients with multiple sclerosis [36].

When clinical signs appear, both macrophages and T lymphocytes invade structures of the brain that are close to periventricular or extraventricular CSF spaces. Preliminary data show that proliferating Ki-67^+^ cells represent less than 5% of the infiltrating cells, indicating that the large infiltrates observed do not result from local cell proliferation only. Variability in the severity and relapsing course of the disease was previously reported in the DA rat model [37,38]. It was shown to correlate with the degree of macrophage infiltration in the brain during the first clinical episode [37]. In our study, all EAE diseased rats develop a first clinical episode with comparable severity, and consistently show tissue infiltration by immune cells including macrophages. We observe a limited heterogeneity in the localization of leucocytes infiltrates, for example in the tissue between the corner of the lateral ventricle anterior horn and the corpus callosum (Figure 4C). Importantly, all infiltrates of either myeloid cells or T lymphocytes, display similar features in the entire set of EAE-diseased animals (both rats and mice). Cells either infiltrate the tissue along a downward CSF-to-tissue density gradient, or alternatively organize around perivascular spaces of the vessels lying in the vicinity of the CSF, sparing the deeper tissue. This again suggests that cells or molecular factors borne by the fluid are crucial determinants for initial tissue infiltration in the forebrain and midbrain, and indicates that perivascular spaces are preferential pathways of cell or proinflammatory factor diffusion from the CSF. This does not preclude a subsequent direct infiltration of immune cells across the endothelium of these vessels once they become activated. Note that neither in rat nor in mouse are tissue infiltrates seen in the subpial layers of the cortices, despite the presence of vessels running at their surfaces. These likely results from the very limited CSF volume and slow CSF circulation over the cerebral surface in rodents whose brain lacks, apart from the rhinal fissure, the dense network of circumvolutions present in that of human and non-human primates.

## Conclusion

This paper extends the spatiotemporal knowledge on the early infiltration of immune cells in the CNS following peripheral inflammation and EAE. It shows in both rat and mouse that leukocytes initially infiltrate distinctive extraventricular CSF-filled compartments of the forebrain and midbrain such as the velum interpositum and ambient cisterns. The involvement of blood-borne myeloid cells in both the immune surveillance of the brain and the effector phase of the neuroinflammatory disease is highlighted. The CPs are not sites of immune cell accumulation during EAE, but may a site of early infiltration of pathogenic immune cells into the CSF. The CSF pleiocytosis and the pattern of cell infiltration in periventricular and pericisternal areas support the implication of the CSF circulatory system in the distribution of immune cells within the forebrain and midbrain during EAE.

## Abbreviations

BBB: Blood-brain barrier; BCSFB: Blood-cerebrospinal fluid barrier; BSA: Bovine serum albumin; CFA: Complete Freund’s adjuvant; CNS: Central nervous system; CP: Choroid plexus; CS: Clinical score; CSF: Cerebrospinal fluid; DA: Dark Agouti; EAE: Experimental autoimmune encephalomyelitis; MOG: Myelin oligodendrocyte glycoprotein; MPO: Myeloperoxidase; MS: Multiple sclerosis; PBS: Phosphate buffer saline; PI: Peripherally inflamed.

## Competing interests

The authors declare that they have no competing interests.

## Authors’ contributions

CS carried out the EAE inductions and follow-up, immunological analysis, and participated in the design of the study. NS participated in the design of the study, experimental work, and the writing of the manuscript. JFGE conceived the study and drafted the manuscript. All authors read and approved the final manuscript.

## Supplementary Material

Additional file 1 Representative drawings of the different areas selected for immunofluorescence analysis. Click here for file

Additional file 2 Time course of the daily weight and clinical score in control, PI, and EAE-diseased animals. Click here for file

Additional file 3 Quantification of immune cells associated with choroid plexuses in control, PI, and EAE-diseased animals. Click here for file

Additional file 4 **Cisternal CD45**^**+**^**infiltrating cells, and perivascular infiltrating cells, are negative for TUNEL staining during the first clinical episode in EAE-diseased rats.** Click here for file

Additional file 5 Schematic distribution of infiltrates in the brain of EAE-diseased animals. Click here for file
