# Supplementary material for: Brain leukocyte infiltration initiated by peripheral inflammation or experimental autoimmune encephalomyelitis occurs through pathways connected to the CSF-filled compartments of the forebrain and midbrain
Source: J Neuroinflammation. 2012 Aug 7;9:187. doi: 10.1186/1742-2094-9-187 (PMC3458946; doi:10.1186/1742-2094-9-187)
Supplement: Additional file 1 — Representative drawings of the different areas selected for immunofluorescence analysis. [file 1742-2094-9-187-S1.pdf]

Additional File 1

Schmitt C, et al: Brain leukocyte infiltration initiated by peripheral inflammation or EAE occurs through pathways connected to the CSF-filled compartments of the forebrain and midbrain.

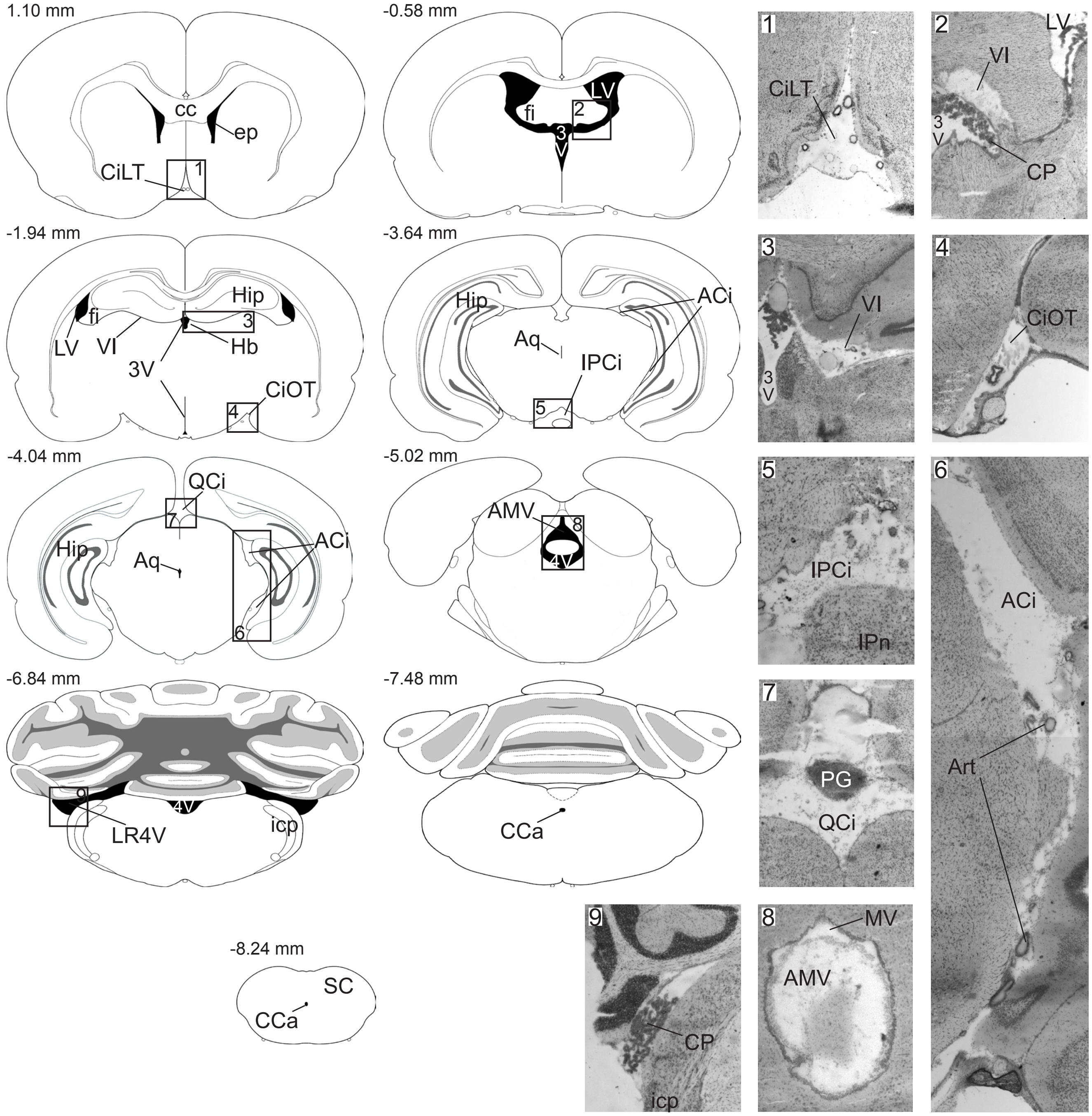

Representative drawings of the different areas selected for immunofluorescence analysis. The bregma position is indicated in the upper left corner of each drawing. Rectangles correspond to cresyl violet stained sections (right) highlighting the different extraventricular CSF-containing compartments.

3V, third ventricle - 4V, fourth ventricle - ACi, ambient cistern - AMV, anterior medullary velum - Aq, aquaduct - Art, arteries - cc, corpus callosum - CCa, central canal - CiLT, cistern of the laminae terminalis - CiOT, cistern of the optic tract - CP, choroid plexus - ep, ependyma - fi, fimbria - Hb, habenula - Hip, hippocampus - icp, inferior cerebellar peduncle - IPCi, interpeduncular cistern - IPn, interpeduncular nucleus - LR4V, lateral recess of 4V - LV, lateral ventricle - MV, mesencephalic ventricle - PG, pineal gland - QCi, quadrigeminal cistern - SC, spinal cord - VI, velum interpositum
