# Supplementary material for: Brain leukocyte infiltration initiated by peripheral inflammation or experimental autoimmune encephalomyelitis occurs through pathways connected to the CSF-filled compartments of the forebrain and midbrain
Source: J Neuroinflammation. 2012 Aug 7;9:187. doi: 10.1186/1742-2094-9-187 (PMC3458946; doi:10.1186/1742-2094-9-187)
Supplement: Additional file 2 — Time course of the daily weight and clinical score in control, PI, and EAE-diseased animals. [file 1742-2094-9-187-S2.pdf]

Additional File 2

Schmitt C, et al: Brain leukocyte infiltration initiated by peripheral inflammation or EAE occurs through pathways connected to the CSF-filled compartments of the forebrain and midbrain.

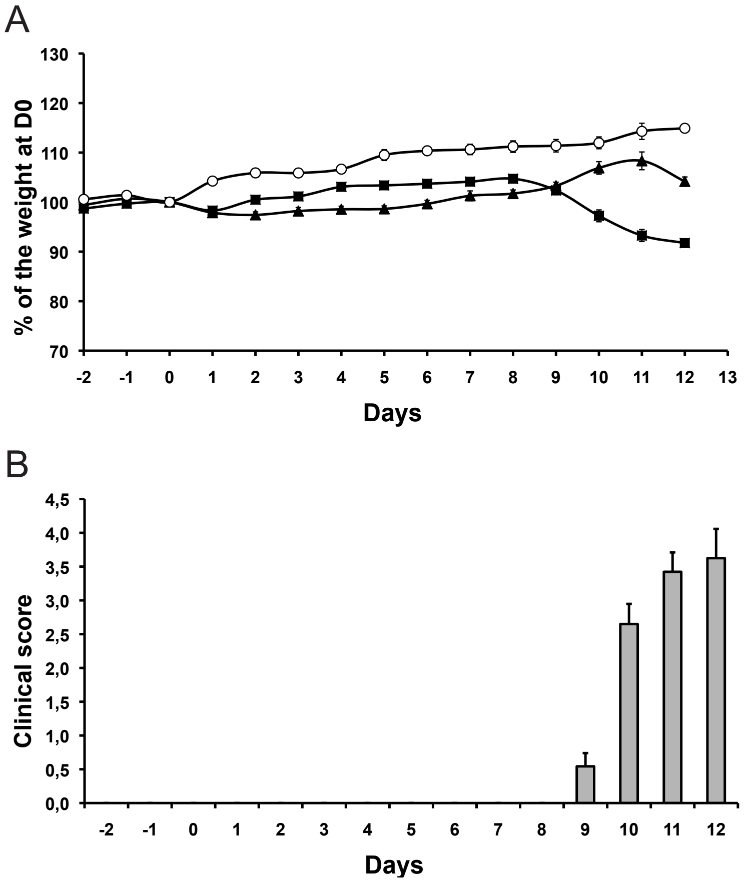

Time course of the daily weight and clinical score in control, PI and EAE-diseased animals. A. Weight of control (open circle), PI (triangle) and EAE-diseased (square) rats. Data are expressed as the mean ± SEM of the percentage of the initial weight of the animal at the time of the induction (D0). Data are combined from 4 different experiments for a total of 93 rats, with a minimum of 5 animals at each time point up to D11. One control, 1 PI and 7 EAE diseased animals were kept up to D12. The weight of both PI and EAE diseased animals is significantly lower than the weight of control animals, from D1 post-treatment, up to D9 (PI), or D11 (EAE). The weight of EAE diseased animals becomes significantly lower than the weight of PI animal from D10 to D12 (all levels of significance: $p<0,05$; Anova followed by post-hoc Tukey-Kramer test). B. Clinical course of EAE in rat. The first neurological signs (onset) appeared on D9 and the peak of the disease occurred on D11-12, matching the period of prominent weight loss reported in A. In mice, a similar weight profile and clinical course was also observed for PI and EAE animals, but the smaller number of animals did not allow a statistical analysis. Neurological signs in EAE-diseased mice were first observed on D8. The peak of the disease lasted for a longer period than in rat, i.e. between D11 and D16, and was also accompanied by a marked weight loss (not shown).
