# Supplementary material for: Brain leukocyte infiltration initiated by peripheral inflammation or experimental autoimmune encephalomyelitis occurs through pathways connected to the CSF-filled compartments of the forebrain and midbrain
Source: J Neuroinflammation. 2012 Aug 7;9:187. doi: 10.1186/1742-2094-9-187 (PMC3458946; doi:10.1186/1742-2094-9-187)
Supplement: Additional file 3 — Quantification of immune cells associated with choroid plexuses in control, PI, and EAE-diseased animals. [file 1742-2094-9-187-S3.pdf]

Additional File 3

Schmitt C, et al: Brain leukocyte infiltration initiated by peripheral inflammation or EAE occurs through pathways connected to the CSF-filled compartments of the forebrain and midbrain.

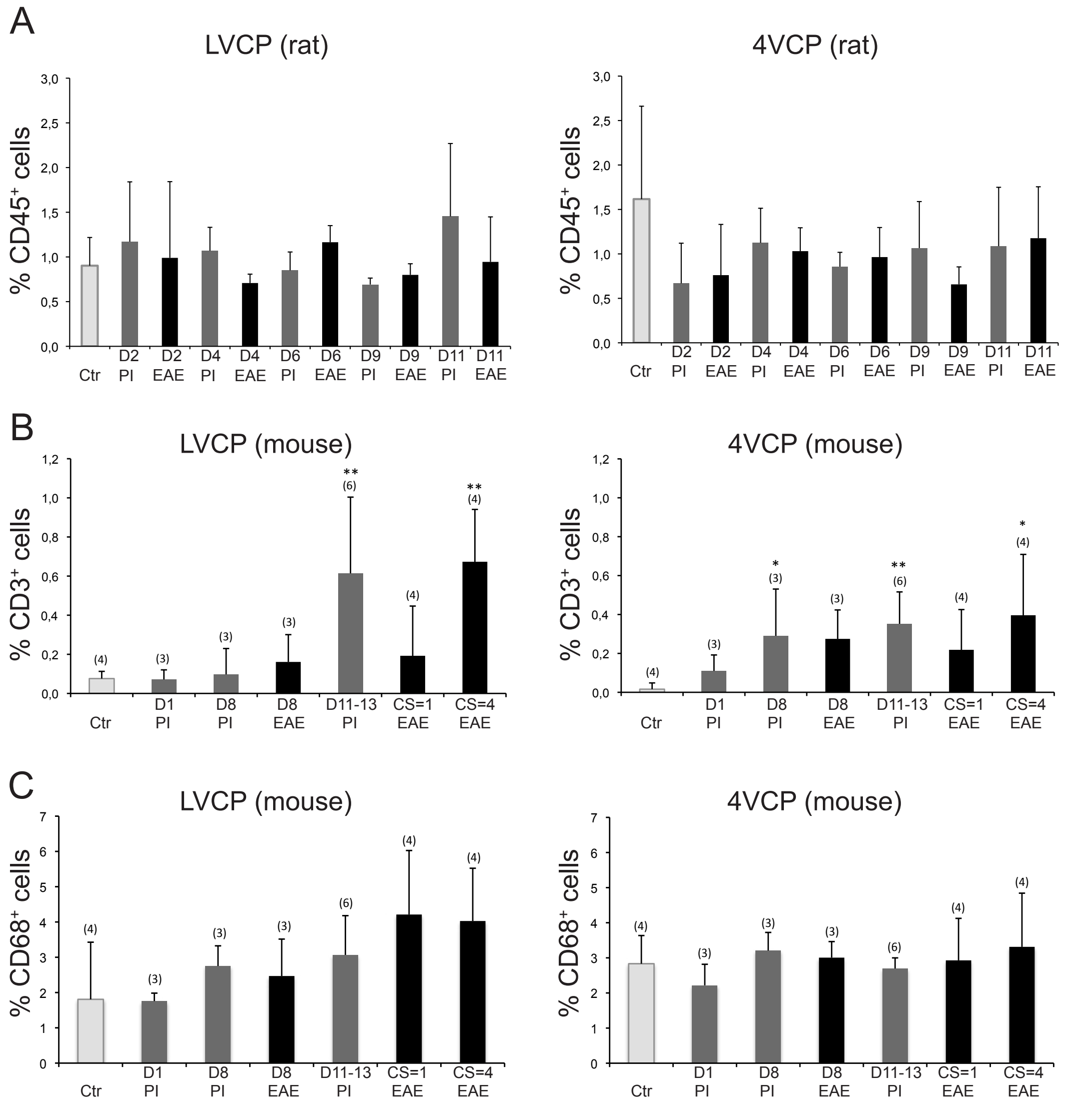

Quantification of immune cells associated with choroid plexuses in control, PI and EAE-diseased animals. A. $CD45^+$ cells associated with lateral (left panel) and fourth (right panel) CP in rat. Data represent mean ± SD, with n=3. B-C. $CD3^+$ and $CD68^+$ cells associated with lateral (left panels) and fourth (right panels) CP respectively, in mice. Ctr: Control, PI: Peripherally inflammed, CS=1: clinical score of 1, CS=4: clinical score of 4, (n). $*p < 0.05$; $**p < 0.01$, statistically different from value in control animals, Dunnett's test.
