# Supplementary material for: Brain leukocyte infiltration initiated by peripheral inflammation or experimental autoimmune encephalomyelitis occurs through pathways connected to the CSF-filled compartments of the forebrain and midbrain
Source: J Neuroinflammation. 2012 Aug 7;9:187. doi: 10.1186/1742-2094-9-187 (PMC3458946; doi:10.1186/1742-2094-9-187)
Supplement: Additional file 4 — Cisternal CD45+infiltrating cells, and perivascular infiltrating cells, are negative for TUNEL staining during the first clinical episode in EAE-diseased rats. [file 1742-2094-9-187-S4.pdf]

Additional File 4

Schmitt C, et al: Brain leukocyte infiltration initiated by peripheral inflammation or EAE occurs through pathways connected to the CSF-filled compartments of the forebrain and midbrain.

Basal cistern **Positive control (DNAse-treated)**

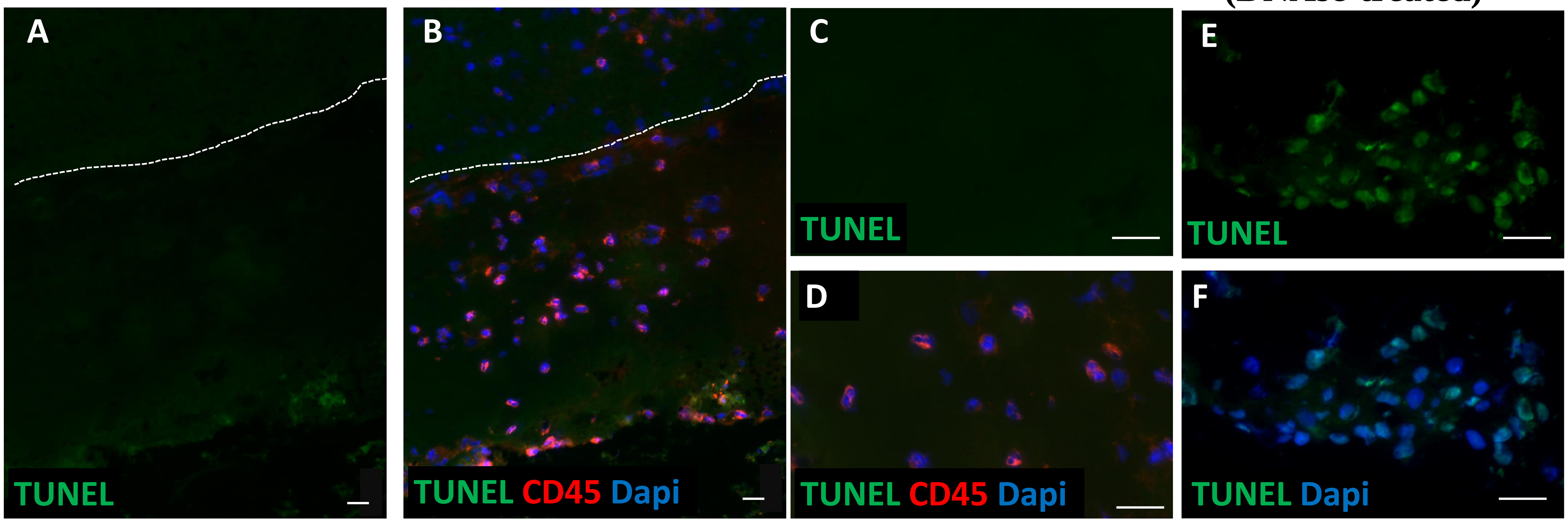

Ambient Cistern **Positive control (DNAse-treated)**

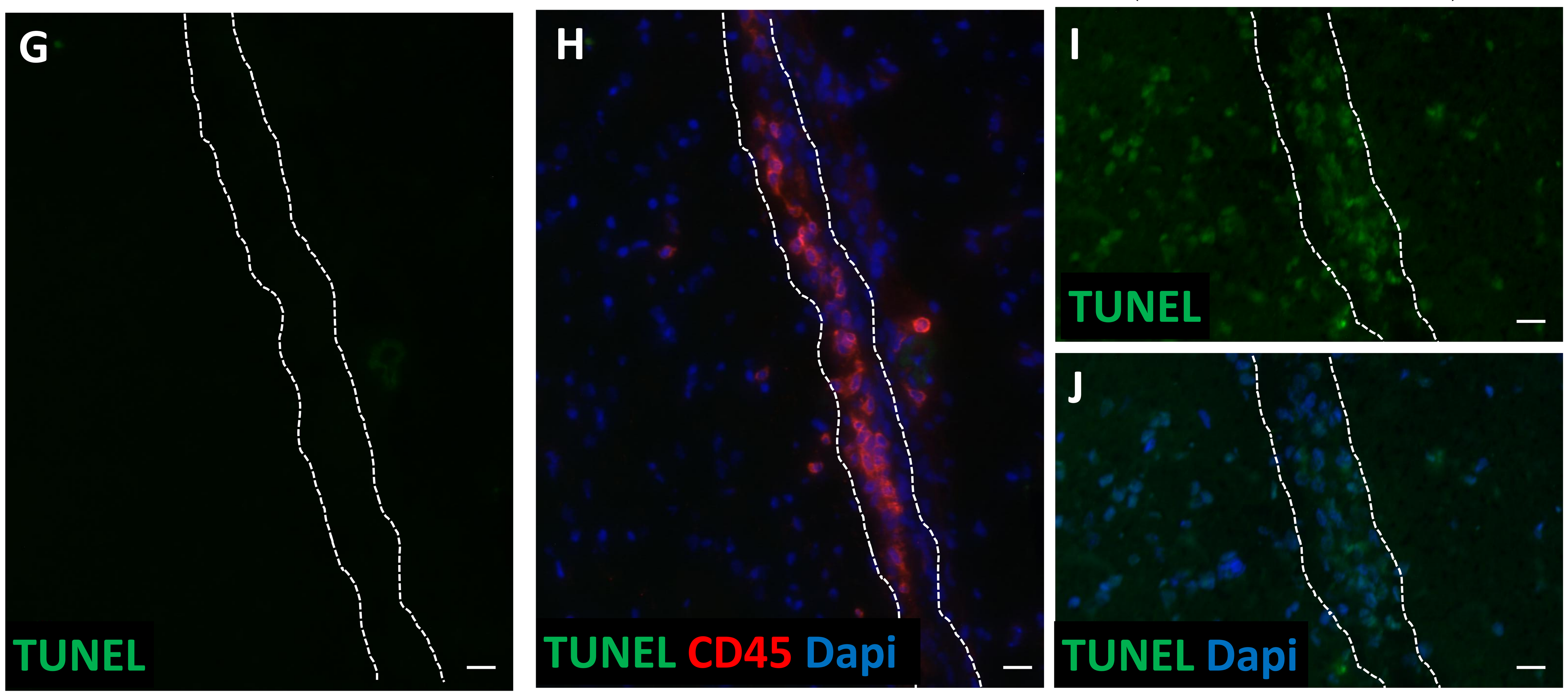

Parenchyma **Positive control (DNAse-treated)**

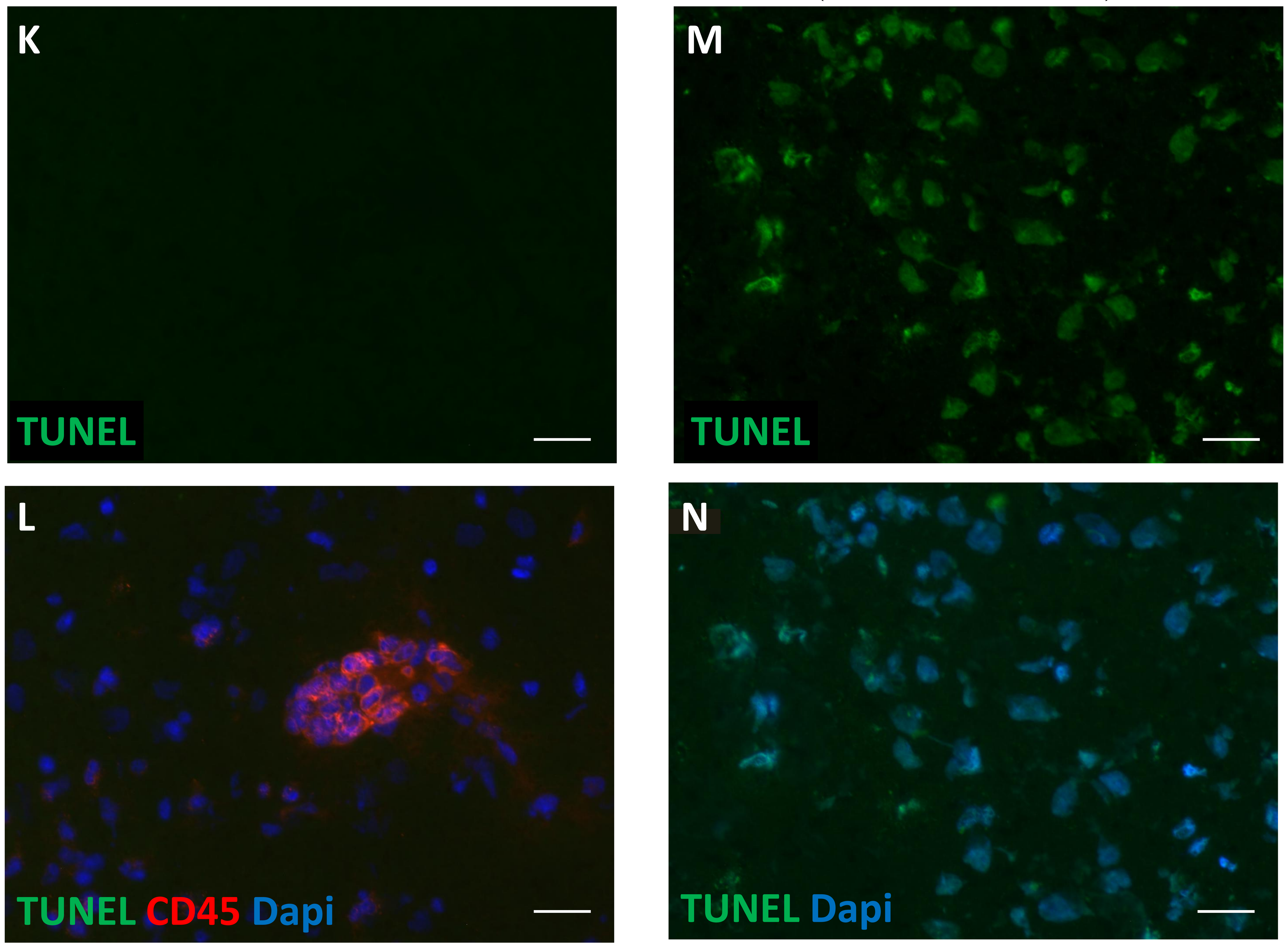

A-F: Infiltrates in the basal cistern of the brain. A,B and C,D (higher magnification): CD45^{+} infiltrates are negative for TUNEL staining. E, F: TUNEL positive control.

G-J: Infiltrates in the ambient cistern. G,H: CD45^{+} infiltrates are negative for TUNEL staining. I,J: TUNEL positive control.

K-N : Infiltrates in brain parenchyma . K,L: CD45^{+} perivascular infiltrates are negative for TUNEL staining. M,N: TUNEL positive control.

Cisternal CD45^{+} infiltrating cells, and perivascular infiltrating cells, are negative for TUNEL staining during the first clinical episode in EAE-diseased rats.

TUNEL positive controls are obtained by treating the sections with DNAse I. Scale bar : 20 µm. Dashed lines delimit the CSF spaces.

.
