# Supplementary material for: Brain leukocyte infiltration initiated by peripheral inflammation or experimental autoimmune encephalomyelitis occurs through pathways connected to the CSF-filled compartments of the forebrain and midbrain
Source: J Neuroinflammation. 2012 Aug 7;9:187. doi: 10.1186/1742-2094-9-187 (PMC3458946; doi:10.1186/1742-2094-9-187)
Supplement: Additional file 5 — Schematic distribution of infiltrates in the brain of EAE-diseased animals. [file 1742-2094-9-187-S5.pdf]

Additional File 5

Schmitt C, et al: Brain leukocyte infiltration initiated by peripheral inflammation or EAE occurs through pathways connected to the CSF-filled compartments of the forebrain and midbrain.

Day 9: first neurological signs

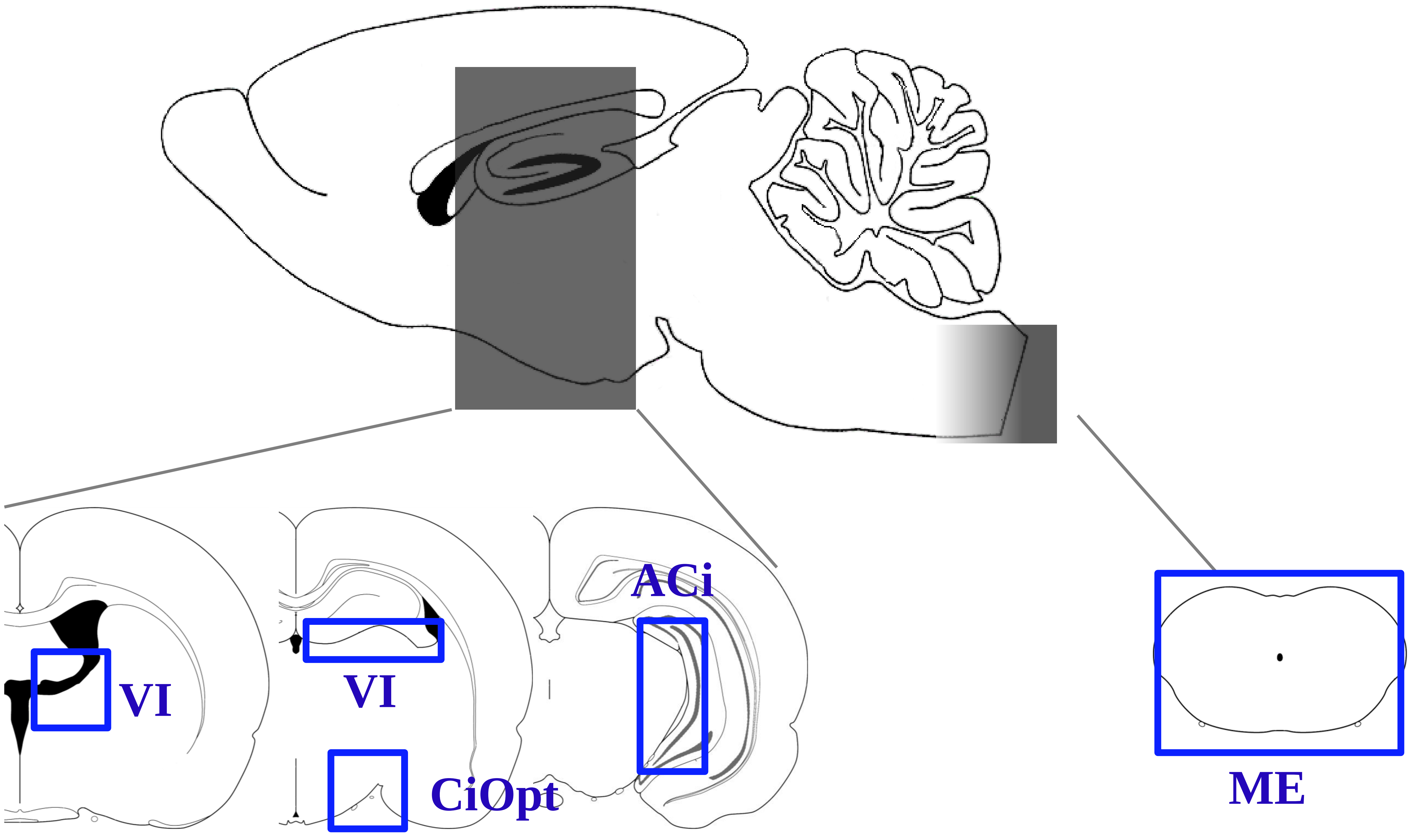

Day 11: hindlimb paralysis

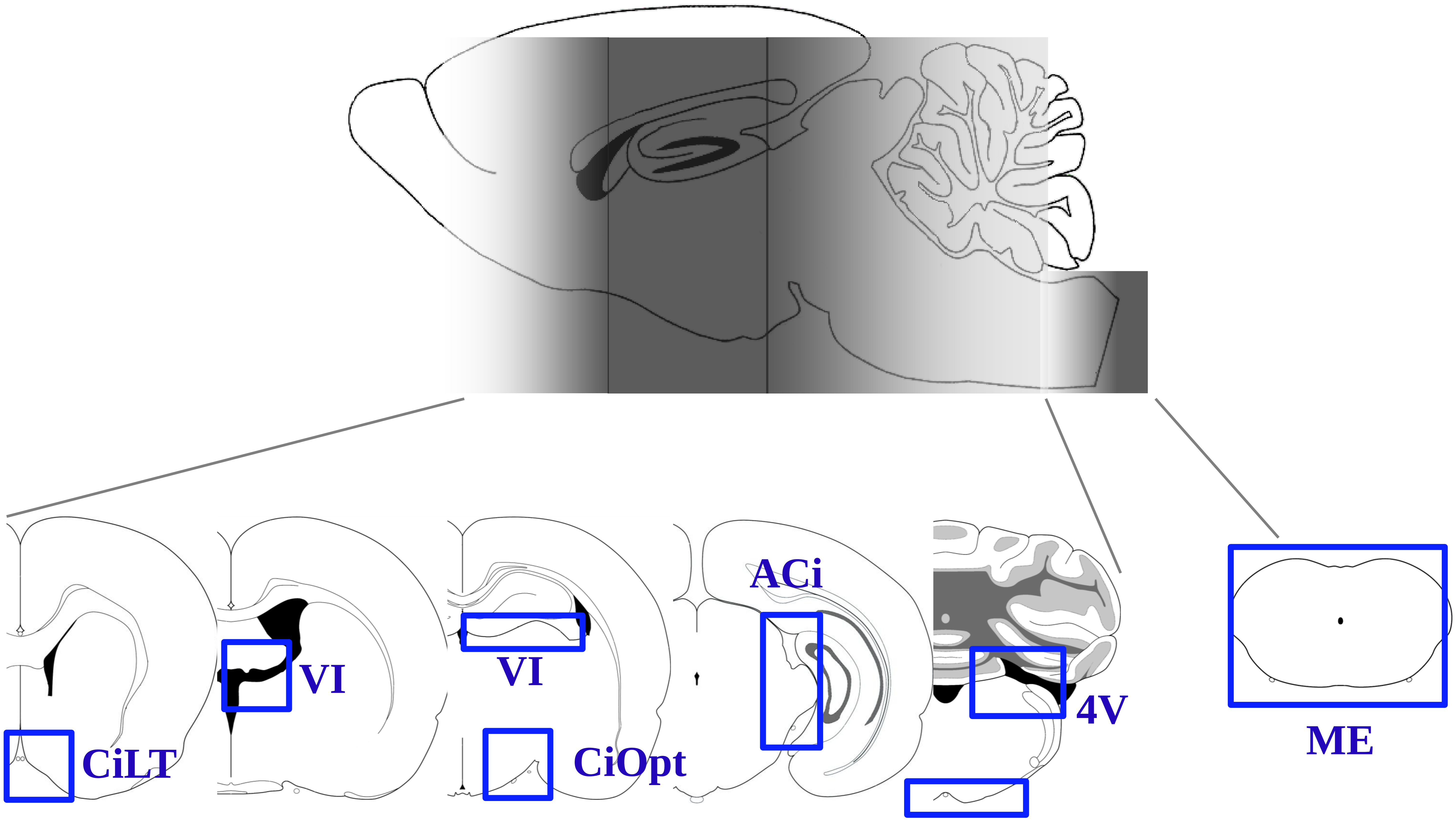

Schematic distribution of infiltrates in the brain of EAE-diseased animals.
A. When the clinical signs appear, infiltrates localize in distinctive areas of the midbrain and forebrain including the velum interpositum space (VI), the tissue in the vicinity of the interventricular junction, the ambient (ACi) and optic (CiOpt) cisterns. They are also present in the spinal cord tissue close to the subarachnoid space (SC). B. At the peak of the disease, the pattern of brain infiltration is wider, extending rostrally to the laminae terminalis (CiLT) and caudally to the fourth ventricular area (4V). Grey zones represent the extent of immune cell infiltration.
